# Supplementary material for: Lossy compression of statistical data using quantum annealer
Source: Sci Rep. 2022 Mar 9;12:3814. doi: 10.1038/s41598-022-07539-z (PMC8907274; doi:10.1038/s41598-022-07539-z)
Supplement: Supplementary file 1 — Supplementary Information. [file 41598_2022_7539_MOESM1_ESM.pdf]

Supplementary Information for
Lossy compression of statistical data using quantum annealer

Boram Yoon[1*], Nga T.T. Nguyen[2†], Chia Cheng Chang[3,4,5‡], and Ermal Rrapaj[4§]

[1]CCS-7, Computer, Computational and Statistical Sciences Division,
Los Alamos National Laboratory,Los Alamos, NM 87545, USA
[2]CCS-3, Computer, Computational and Statistical Sciences Division,
Los Alamos National Laboratory,Los Alamos, NM 87545, USA
[3]RIKEN iTHEMS, Wako, Saitama 351-0198, Japan
[4]Department of Physics, University of California, Berkeley, California 94720, USA
[5]Nuclear Science Division, Lawrence Berkeley National Laboratory,
Berkeley, California 94720, USA

1 Performance of Simulated Annealing Sampler

In this section, we evaluate the quality of the D-Wave's simulated annealing sampler. Consider a problem finding N_q binary coefficients $\boldsymbol{a} \in \{0,1\}^{N_q}$ of the positive powers of $r^{-1} < 1$ that precisely reconstruct a uniform random number $z \in [0, 1)$ by minimizing the following reconstruction error

$$E = \left| z - \frac{1}{R} \sum_{n=1}^{N_q} a_n r^{-n} \right| \qquad \text{where } R \equiv \sum_{n=1}^{\infty} r^{-n} \,. \tag{1}$$

The optimization problem can be converted into QUBO form of the main text Eq. (3) by the taking the transformation given in the main Eq. (4) after replacing $\boldsymbol{\phi}$ and $\mathbf{X}$ with the vector of $\{r^{-n}/R\}$ and z, respectably,

$$\boldsymbol{\phi} \to \begin{bmatrix} r^{-1}/R \\ r^{-2}/R \\ r^{-3}/R \\ \vdots \\ r^{-N_q}/R \end{bmatrix}, \qquad \mathbf{X} \to z \,.$$

*boram@lanl.gov
†nga.nguyen@lanl.gov
‡chiacheng.chang@riken.jp
§ermalrrapaj@berkeley.edu

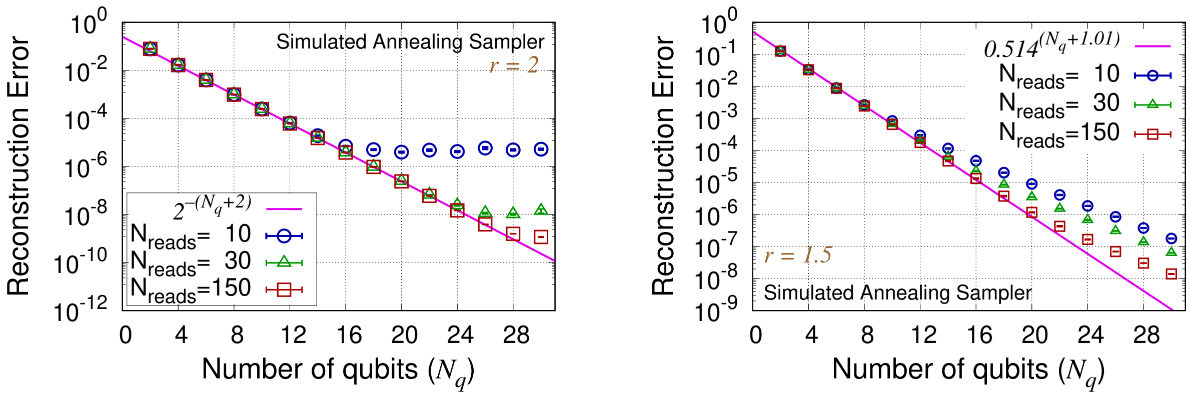

Figure S1: Average reconstruction error defined in the main text Eq. (17) for different number of reads (10, 30, and 150) of the simulated annealing sampler for $r = 2$ (left) and $r = 1.5$ (right). The expected average reconstruction error of the ideal QUBO solver is plotted as a magenta line.

We solve the QUBO problem using D-Wave's simulated annealing sampler on a classical computer by taking the minimum energy solution from the three different choices of $num\_reads = 10, 30$, and 150, keeping other sampler parameters set to default, for 10^5 random numbers of r and calculate the average value of the reconstruction error E. The study is done at two different values of $r = 2$ and 1.5. When $r = 2$, it becomes a simple decimal to binary conversion problem, whose optimal solution is known. The expected value of the average reconstruction error for an ideal QUBO solver for $N_q \gg 1$ is $2^{-(N_q+2)}$. For $r = 1.5$, we calculate the empirical average reconstruction error of an ideal QUBO solver by fitting the average values of the reconstruction errors obtained using the exact solver implemented in the D-Wave Ocean library, which finds the minimum energy solution by comparing the energies of all possible solutions. The two free parameters of the fitting functional form a^{N_q+b} are determined to be $a = 0.514(2)$ and $b = 1.01(6)$ from the 7 data points at $N_q = 8, 10, 12, \ldots, 20$ with the $\chi^2/\text{dof} = 1.26$.

Figure S1 shows that a larger number of reads makes the reconstruction error smaller, the problems with the larger number of qubits require the larger number of reads to make the solution close to the exact solution. Results show that the simulated annealing sampler with num_reads=150 gives the solution close to the ground-energy up to $N_q \approx 20$, but it may depend on the problem, as demonstrated by the difference between the $r = 1.5$ and $r = 2$ cases.
